# Persistent current in a correlated quantum ring with electron-phonon interaction in the presence of Rashba interaction and Aharonov-Bohm flux

**DOI:** 10.1038/srep20056

**Published:** 2016-02-01

**Authors:** P. J. Monisha, I. V. Sankar, Shreekantha Sil, Ashok Chatterjee

**Affiliations:** 1 School of Physics, University of Hyderabad, Hyderabad 500046, India; 2 Department of Physics, Visva-Bharati-Santiniketan, West Bengal-731235, India

## Abstract

Persistent current in a correlated quantum ring threaded by an Aharonov-Bohm flux is studied in the presence of electron-phonon interactions and Rashba spin-orbit coupling. The quantum ring is modeled by the Holstein-Hubbard-Rashba Hamiltonian and the energy is calculated by performing the conventional Lang-Firsov transformation followed by the diagonalization of the effective Hamiltonian within a mean-field approximation. The effects of Aharonov-Bohm flux, temperature, spin-orbit and electron-phonon interactions on the persistent current are investigated. It is shown that the electron-phonon interactions reduce the persistent current, while the Rashba coupling enhances it. It is also shown that temperature smoothens the persistent current curve. The effect of chemical potential on the persistent current is also studied.

The existence of a persistent current (PC) in a normal metal ring was first proposed by Buttiker, Imry and Landauer^1^. Cheung *et al.*^2^ have studied the effects of temperature, chemical potential and randomness on PC in strictly one-dimensional (1D) normal rings. Several theoretical studies^3^,^4^,^5^,^6^,^7^ have been subsequently carried out on PC in mesoscopic systems. Since the energy would be periodic in the flux, one expects the PC to show the similar behavior. With the advent of nano-fabrication techniques, several experimental investigations have been made to confirm the existence^8^,^9^ and the periodicity^10^,^11^,^12^,^13^,^14^ of PC in semiconductor quantum rings (QRs). The periodicity of PC in a finite ring can be shown using continuum or discrete models^15^. The period is found to be 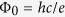 for non-interacting spinless electrons. The most useful model to study PC is the Hubbard model in which the ring consists of discrete lattice sites and the electrons can hop from one site to another. Several works^16^,^17^,^18^,^19^ have been carried out on the Hubbard ring to understand the magnetic response and the behavior of PC. But most of them have neglected the electron-phonon (*e-p*) interaction which can actually play quite an important role in the low-dimensional systems. The effect of *e-p* interaction on PC can be captured by considering the Holstein-Hubbard (HH) model^20^,^21^. Another important interaction that has come to light in the context of nanosystems in recent years is the spin-orbit (SO) interaction which is at the heart of the emerging field of spintronics. New devices are being contemplated which would use the spin degrees freedom instead of charge. There can be two kinds of SO interactions in solids. One originates due to the structural inversion asymmetry which is known as the Rashba spin-orbit (RSO) interaction and the other is due to the bulk inversion asymmetry which is called as the Dresselhaus spin-orbit (DSO) interaction. The effects of SO interaction^22^,^23^,^24^ are found to be pronounced in QR’s. By tuning the external electric field the electron spin can be controlled and consequently, the Rashba effect can be manipulated, which is precisely the idea behind spintronics^25^. In the present paper we shall study the effects of RSO interaction on PC in a 1D HH ring threaded by an Aharonov-Bohm (AB) flux. Since the number of electrons in a QR also changes the magnitude and phase of PC^2^, the chemical potential is expected to have an interesting effect on PC. As the temperature increases, the electrons may occupy higher energy levels that are close and can have opposite currents and therefore, as a net result, the higher positive and negative contributions to PC may cancel out. Buttiker has indeed observed a decrease in the amplitude of the PC with temperature^26^. We shall therefore study the effects of chemical potential and temperature as well on PC in a 1D HH ring in the presence of RSO interaction.

## Theoretical Formalism

The Hamiltonian for a HH ring threaded by a magnetic flux 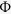 can be written in the presence of RSO interaction as


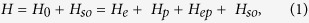


where


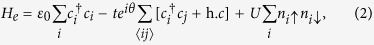



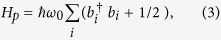



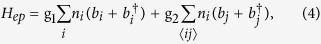



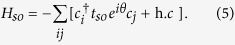


Eq. 2 represents the electronic Hamiltonian 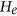 which consists of three terms. The first term represents the site energy, 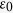 being the site energy, 
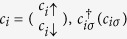
 being the creation (annihilation) operator for an electron at site *i* with spin *σ*, and 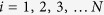, *N* being the total number of sites in the system. The second term describes the hopping term where *t* is the hopping integral between the nearest**-**neighbour (NN) sites, 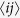 denotes that the summation is to be performed over NN sites *i* and *j* and 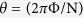 is the AB phase due to the magnetic flux 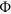, which is an integral multiple of the elementary flux quantum 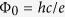. The third term is the onsite repulsive electron-electron (*e-e*) Coulomb interaction where *U* measures the strength of the interaction and 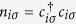 is the number operator for the electrons at site *i* with spin *σ*. Eq. 3 gives the unperturbed phonon Hamiltonian where 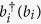 is the creation (annihilation) operator for a phonon with dispersionless frequency 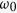 at site *i*. Eq. 4 represents the onsite and NN *e-p* interactions with 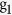 and 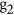 measuring the respective coupling constants. Thus 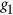 measures the strength of the interaction of an electron with the phonons at the *i*-th site, whereas 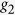 gives the strength of the interaction of an electron at the *i*-th site with the phonons at the 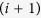-th site. The value of 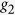 is in general expected to be smaller than that of 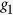 and typically for a real material one may take the value of 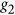 about one order less than that of 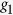. In general, an electron is supposed to interact with phonons at all sites. But we restrict our study of *e-p* interaction up to NN terms assuming that beyond NN’s, interactions will be small enough to be ignored. [If the onsite *e-p* interaction is so strong that the electron gets trapped in a deep potential well created at the *i*-th site, then its interaction with the NN phonons will be very small. In such cases the effective NN *e-p* interaction can be neglected]. In real systems the effects of 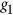 and 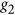 manifest themselves through the localization-delocalization transition. Finally, Eq. 5 describes the SO interaction with


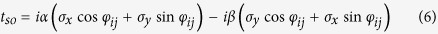


where 
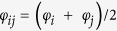
 and 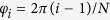 so 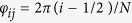, 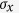 and 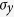 are the Pauli spin matrices. And 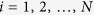 is the site index along the azimuthal direction 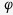 of the ring.

In the present problem we are interested in RSO interaction only and so we take 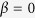. We first perform a Lang-Firsov transformation (LFT) with a generator 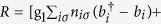 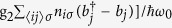. LFT is a displaced oscillator (also called a coherent state) transformation and its purpose is to eliminate the phonons to obtain an effective electronic Hamiltonian. Performing a LFT physically means assuming a coherent state for phonons where the coherence strength is determined by the electron density. The LFT works well in the strong-coupling limit. Next we employ a unitary transformation with the matrix


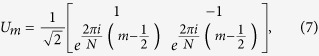


so that the transformed Hamionian reads


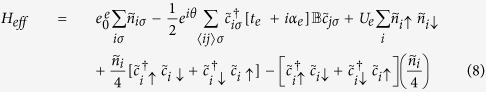


where


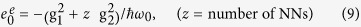



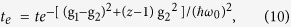



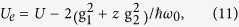



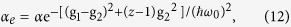



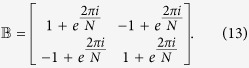



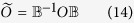


We now use a mean-field approximation (MFA) to deal with the *e*-*e* interaction. This approximation neglects the fluctuations and is known to be a meaningful approximation if the correlation is not strong. Using MFA, we get after some algebra^27^


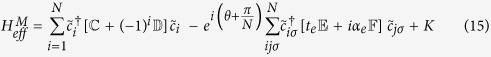


where,


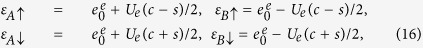



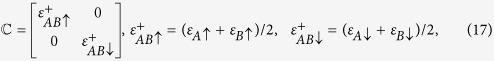



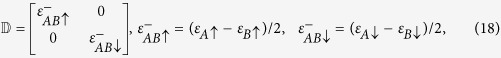



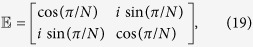



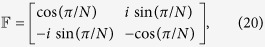



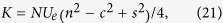



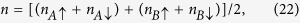



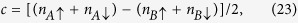



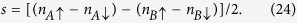


Using the Fourier transform: 
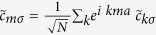
, where *a* is the lattice constant and redefining 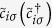 as 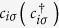 and 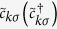 as 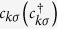 and separating the Hamiltonian into even and odd sited terms, we obtain after some rearrangement of terms


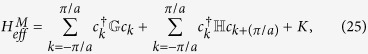


where


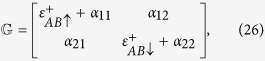



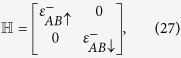



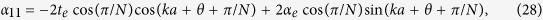



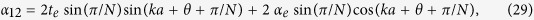



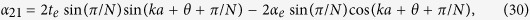



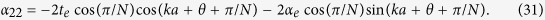


We shall work in the reduced zone scheme i. e., we choose *k* to lie in the range: 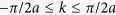. In this scheme, the matrix elements 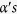 can be written as: 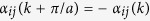. The effective mean-field Hamiltonian can now be written as


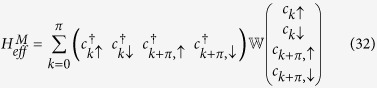


where


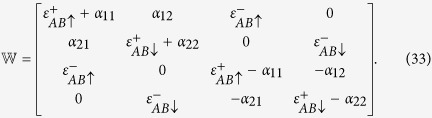


The exact numerical diagonalization of 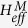 yields four energies 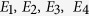 and the four distribution functions *f*(*E*_1_), *f*(*E*_2_), *f*(*E*_3_), *f*(*E*_4_), where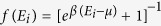. The GS energy is now given by


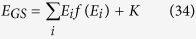


and the PC 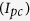 can be calculated from the relation


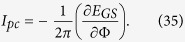


## Numerical results and Discussions

**Figure 1:**
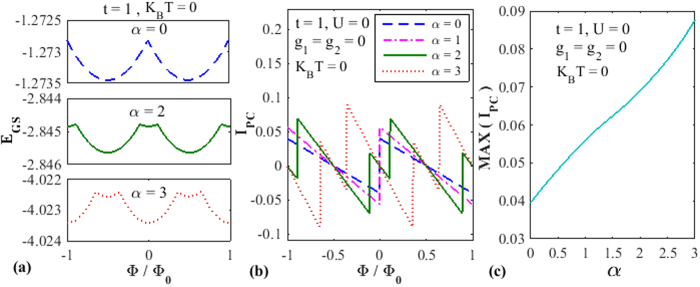
The GS energy and PC in the presence of SO interaction for *U* = g_1_ = g_2_ = 0. (**a**) The GS energy as a function of the flux 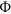 for different *α*. (**b**) Persistent current 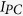 as a function of 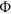 for different *α*. **(c)** Variation of 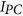 as a function of *α*.

For the sake of convenience, we set 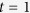 and measure all energies in units of 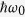. In Fig. 1(a), we plot the GS energy 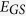 as a function of the flux 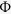 for various values of *α* in the absence of all other interactions. We can see that the GS energy increases with *α*. The periodicity of the energy with 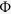 is also clearly evident. In Fig. 1(b) we plot PC vs. 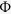 for different values of *α*. The RSO interaction clearly enhances 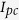. Also, the phase of PC changes, when *α* exceeds a critical value 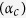. In the present case, 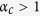. The variation of 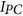 as a function of *α* is explicitly shown in Fig. 1(c). 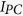 increases with *α* monotonically, though its derivative can have a more interesting behavior.

**Figure 2:**
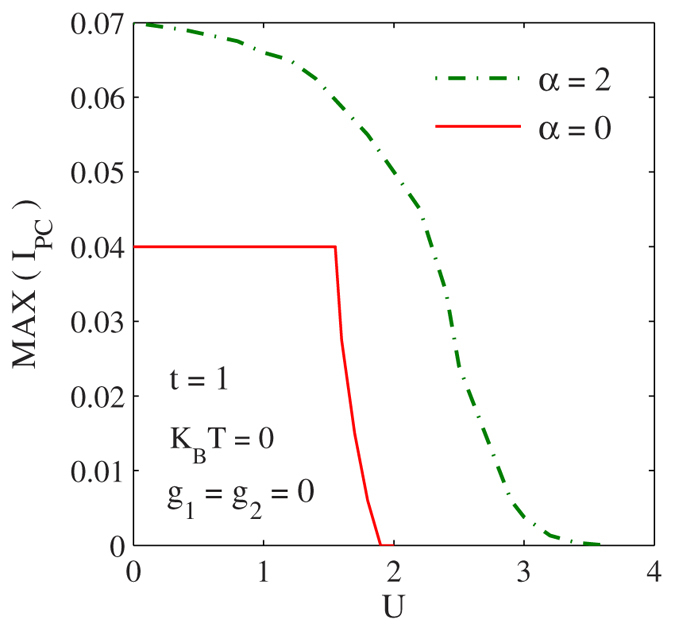
The effect of *e-e* interaction on the PC. *I*_*PC*_ as a function of *U* for *α* = 2 and *α* = 0.

Figure 2 shows the behaviour of PC as a function of *U* with and without RSO interaction. The MFA employed here may be considered to be a reasonable approximation since we have studied the effect of *U* from *U* = 0 to *U* = 4 which lies in the weak correlation regime since 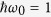 is set as the energy scale. The solid line describes the behavior for 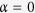 and the dashed-dotted line for 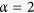. One can easily notice that PC decreases as *U* increases. The explanation is quite simple. As *U* increases, the electrons experience a larger onsite repulsion and thus find it more difficult to go from one site to another. This reduces PC. In the absence of RSO interaction, there seems to exist some critical value of *U* 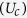 below which PC remains constant and unaffected by *U*. This implies that the effective hopping parameter 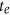 remains predominant over *U* below *U*_*c*_ and thus *U* does not play any significant role. Above *U*_*c*_, PC dies out extremely sharply. In the presence of RSO interaction, however, there is a qualitative difference in the behavior of PC as a function of *U*. The figure shows the behavior for 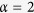. It is evident that, though even now, the decrease of PC with increasing *U* is quite rapid, it is smooth and there is no indication of existence of any critical value of *U*. One possible explanation for this behavior may be the following. In the presence of the Rashba interaction, the PC undergoes a significant enhancement as can be seen from Fig. 1(c) and the correlation effect also increases because of a second-order contribution from the RSO interaction. Because of the higher effective *U*, even at small *U*, the correlation plays an important role and consequently PC decreases with *U* from *U* = 0 itself and thus shows a smooth behavior as a function of *U*. Use of a better method to include the quantum fluctuations may make the PC vs. *U* curve smooth even in the case of 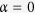 and rule out the existence of any critical 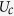. A final answer on this issue requires more critical investigations.

**Figure 3:**
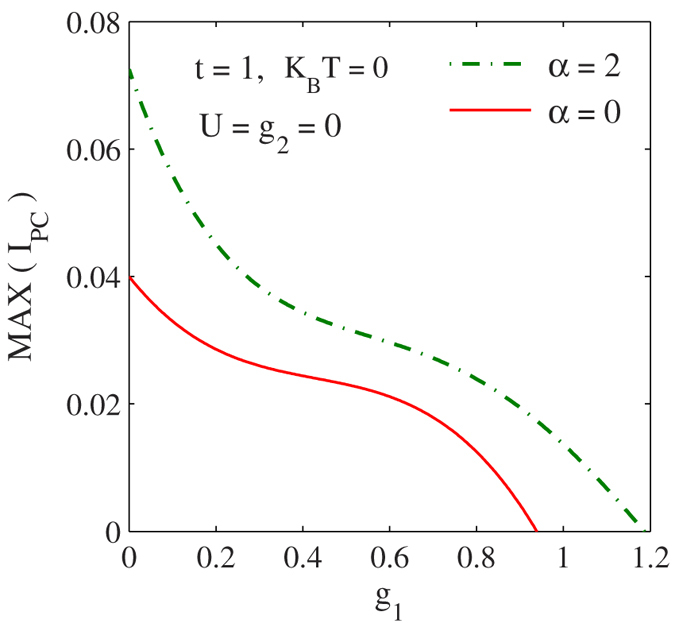
The effect of *e-p* interaction on the PC. *I*_*PC*_ vs. 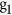 for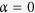 and 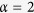 (with *U* = 0 = g_2_).

Next we look into the effects of onsite and NN *e-p* interactions on PC. In Fig. 3 we plot PC as a function of g_1_. It is evident that PC decreases as 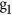 increases. This reduction in PC is understandable. Since 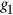 gives the strength of the onsite *e-p* interaction, as 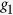 is increased, the *e-p* interaction will distort the lattice more around that site leading to a deeper polarization potential for the electron causing electron self-trapping or localization at that site^28^,^29^,^30^. This will inhibit conduction. The gradient of the curve is however not monotonic which may have some interesting physical implications. One may also notice that the resistive effect of the *e-p* interaction is more pronounced than that of the *e-e* interaction.

**Figure 4:**
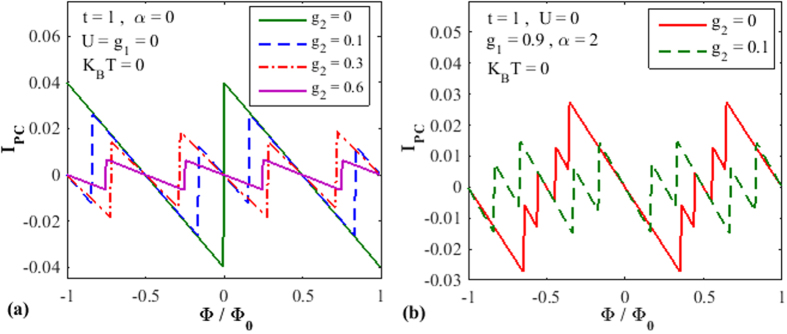
The effect of NN *e-p* interaction on the PC. (**a**) *I*_*pc*_ vs. 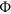 for different values of 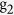 with 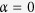. (**b**) PC vs 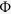 for 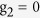 and 0.1 with 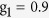.

In Fig. 4(a), we wish to study the effect of NN *e-p* interaction on PC keeping 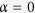. So we plot 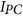 vs. 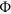 for several values of g_2_. To see the sole effect of g_2_ we first study the case with 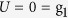. We find that the effect of 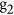 on PC is stronger that of that of g_1_, which is clearly suggested by Eq. 10. According to Eq. 10, 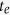 contains an additional Hoslstein reduction factor solely dependent on g_2_. Figure 4(a) also shows that the periodicity of PC decreases with increasing g_2_. The behavior is qualitatively similar (not shown here) even with g_1_, but again the effect of g_2_ is stronger. In Fig. 4(b) we show PC vs. 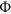 for 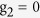 and 0.1 in the presence of onsite *e-p* interaction 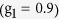. Of course, the reduction in PC is now more pronounced and furthermore the periodicity also decreases. We do not plot PC vs. 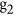 because the behavior is infested with a lot of fluctuations.

**Figure 5:**
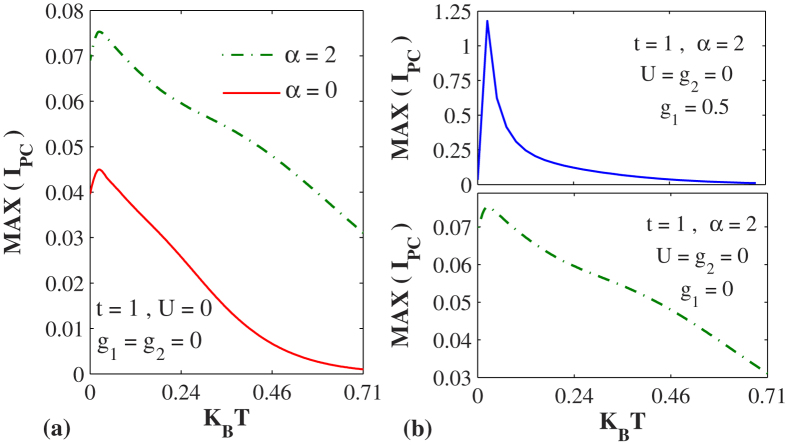
The effect of temperature on the PC. (**a**) *I*_*pc*_ as a function of temperature for 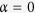 and 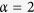. (**b**) 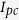 as a function of temperature for different values of 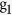 with *α* = 2.

The effect of temperature on PC is plotted in Fig. 5(a) for both 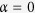 and 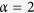 and it is clear that as the temperature increases, PC decreases in both cases as our commonplace notion would justify. The exact numerical behavior is however a little more complicated eluding any simple explanation. As established earlier, PC is larger in the presence of the RSO interaction. Interestingly enough, PC develops a peak at very low temperature. In Fig. 5(b) we plot PC vs. temperature in the presence of RSO interaction for 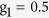 and compare with the graph for 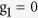. It is evidently clear that in the presence the *e-p* interaction, the peak in PC becomes sharper and acquires a greater value. This happens because as *e-p* interaction increases, polaronic quasiparicle weight increases leading to a sharper peak in the PC.

**Figure 6:**
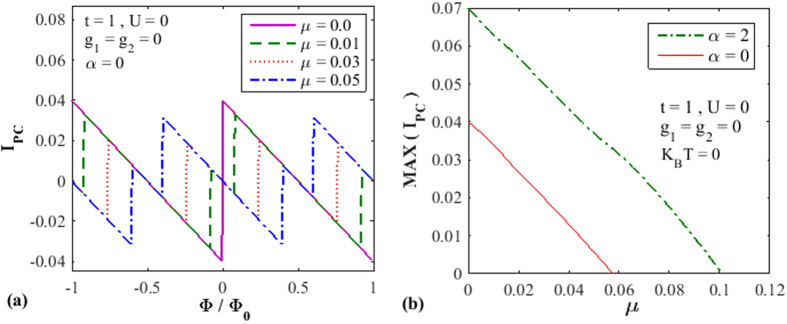
The effect of chemical potential on the PC. (**a**) The persistent current as a function of flux for different *μ*. (**b**) PC vs *μ* for 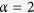 and *α* = 0.

Finally we wish to study the effect of chemical potential *μ* on PC. In Fig. 6(a), we plot PC as a function of 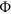 for different values of *μ*. As expected, the magnitude and the phase of PC change with *μ*. Direct dependence of PC on *μ* is shown in Fig. 6(b) both in the presence and absence of the RSO interaction. In both cases, PC decreases with increasing *μ*, the values of PC being greater for the 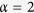.

## Conclusions

In this work, the effect of RSO interaction on PC is studied in a one-dimensional Holstein-Hubbard ring threaded by an Aharonov-Bohm flux. First, the phonon degrees of freedom are eliminated by performing the conventional Lang-Firsov transformation and then the spin-dependence is removed by performing another unitary transformation. The effective electronic Hamiltonian is finally diagonalized by using a mean-field Hartree-Fock approximation and PC is calculated by differentiating the GS energy with respect to the flux. We show that the magnitude of PC is enhanced as we switch on the RSO interaction *α*. Also, for large values of *α* 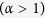, the phase of PC is observed to change. We notice that both the *e-e* and *e-p* interactions reduce the value of PC. We also observe that the NN *e-p* interaction has a stronger effect on PC than the onsite *e-p* interaction has. We furthermore show that PC decreases with temperature and in the presence of *e-p* interaction develops a sharp peak at a low temperature. We finally show that the magnitude of PC decreases with increasing chemical potential and its phase also changes (as the number of particles change).

## Additional Information

**How to cite this article**: Monisha, P. J. *et al.* Persistent current in a correlated quantum ring with electron-phonon interaction in the presence of Rashba interaction and Aharonov-Bohm flux. *Sci. Rep.* **6**, 20056; doi: 10.1038/srep20056 (2016).

## References

[b1] Büttiker M., Imry Y. & Landauer R. Josephson behavior in small normal one-dimensional rings. Phys. Lett. A 96, 365–367 (1983).

[b2] Cheung H.-F., Gefen Y., Riedel E. K. & Shih W.-H. Persistent currents in small one-dimensional metal rings. Phys. Rev. B 37, 6050–6062 (1988). 10.1103/physrevb.37.6050 9943835

[b3] Altshuler B. L., Gefen Y. & Imry Y. Persistent differences between canonical and grand canonical averages in mesoscopic ensembles: Large paramagnetic orbital susceptibilities. Phys. Rev. Lett. 66, 88–91 (1991). 10043149 10.1103/PhysRevLett.66.88

[b4] von Oppen F. & Riedel E. K. Average persistent current in a mesoscopic ring. Phys. Rev. Lett. 66, 84–87 (1991). 10043148 10.1103/PhysRevLett.66.84

[b5] Abraham M. & Berkovits R. Persistent currents in an interacting 1D disordered ring: Manifestations of the Mott-Hubbard transition. Phys. Rev. Lett. 70, 1509–1512 (1993). 10053310 10.1103/PhysRevLett.70.1509

[b6] Giamarchi T. & Shastry B. S. Persistent currents in a one-dimensional ring for a disordered Hubbard model. Phys. Rev. B 5, 10915–10922 (1995). 10.1103/physrevb.51.10915 9977791

[b7] Castelano L. K., Hai G.-Q., Partoens B. & Peeters F. M. Control of the persistent currents in two interacting quantum rings through the Coulomb interaction and interring tunneling. Phys. Rev. B 78, 195315–195315-4 (2008).

[b8] Lévy L. P., Dolan G., Dunsmuir J. & Bouchiat H. Magnetization of mesoscopic copper rings: evidence for persistent currents. Phys. Rev. Lett. 64, 2074–2077 (1990). 10041570 10.1103/PhysRevLett.64.2074

[b9] Chadrasekhar V. *et al.* Magnetic response of a single, isolated gold loop. Phys. Rev. Lett. 67, 3578–3581 (1991). 10044771 10.1103/PhysRevLett.67.3578

[b10] Timp G. *et al.* Suppression of the Aharonov-Bohm effect in the quantized Hall regime. Phys. Rev. B 39, 6227–6230 (1991). 10.1103/physrevb.39.6227 9949054

[b11] Mailly D., Chapelier C. & Benoit A. Experimental observation of persistent currents in GaAs-AlGaAs single loop. Phys. Rev. Lett. 70, 2020–2024 (1993). 10053445 10.1103/PhysRevLett.70.2020

[b12] Liu J. *et al.* Correlations between Aharonov-Bohm effects and one-dimensional subband populations in GaAs/Al_x_Ga_1−x_As rings. Phys. Rev. B 48, 15148–15157 (1993). 10.1103/physrevb.48.15148 10008049

[b13] Jariwala E. M. Q., Mohanty P., Ketchen M. B. & Webb R. A. Diamagnetic Persistent Current in Diffusive Normal-Metal Rings. Phys. Rev. Lett. 86, 1594–1597 (2001). 11290201 10.1103/PhysRevLett.86.1594

[b14] Deblock R., Bel R., Reulet B., Bouchiat V. & Mailly D. Diamagnetic Orbital Response of Mesoscopic Silver Rings. Phys. Rev. Lett. 89, 206803-1–206803-4 (2002). 12443497 10.1103/PhysRevLett.89.206803

[b15] Viefers S., Koskinen P., Singha Deo P. & Manninen M. Quantum rings for beginners: energy spectra and persistent currents. Physica E 21, 1–35 (2004).

[b16] Gupta S., Sil S. & Bhattacharyya B. Half-filled Hubbard ring with alternating site potentials in a magnetic field. Phys. Lett. A 324, 494–500 (2004).

[b17] Maiti S. K., Chowdhury J. & Karmakar S. N. Strange behavior of persistent currents in small Hubbard rings. Phys. Lett. A 332, 497–502 (2004).

[b18] Wei B.-B., Gu S.-J. & Lin H.-Q. Persistent currents in the one-dimensional mesoscopic Hubbard ring. J. Phys.: Condens. Matter 20, 395209–395205 (2008).

[b19] Maiti S. K. Magnetic response in mesoscopic Hubbard rings: A mean field study. Solid State Commun. 150, 2212–2217 (2010).

[b20] Das A. N. & Sil S. A study of the polaronic band width and the small-to-large-polaron transition in a many-polaron system. J. Phys.: Condens. Matter 5, 8265–8276 (1993).

[b21] Takada Y. & Chatterjee A. Possibility of a metallic phase in the charge-density-wave–spin-density-wave crossover region in the one-dimensional Hubbard-Holstein model at half filling. Phys. Rev. B 67, 081102-1–081102-4(R) (2003).

[b22] Koga T., Nitta J., Akazaki T. & Takayanagi H. Rashba Spin-Orbit Coupling Probed by the Weak Antilocalization Analysis in InAlAs*/*InGaAs*/*InAlAs Quantum Wells as a Function of Quantum Well Asymmetry. Phys. Rev. Lett. 89, 046801-1–046801-4 (2002). 12144493 10.1103/PhysRevLett.89.046801

[b23] Premper J., Trautmann M., Henk J. & Bruno P. Spin-orbit splitting in an anisotropic two-dimensional electron gas. Phys. Rev. B 76, 073310-1–073310-4 (2007).

[b24] Maiti S. K., Dey M., Sil S., Chakrabarti A. & Karmakar S. N. Magneto-transport in a mesoscopic ring with Rashba and Dresselhaus spin-orbit interactions. Europhys. Lett. 95, 57008-p1–57008-p6 (2011).

[b25] Bellucci S. & Onorato P. Crossover from the ballistic to the resonant tunneling transport for an ideal one-dimensional quantum ring with spin-orbit interaction. Phys. Rev. B 78, 235312-1–235312-6 (2008).

[b26] Büttiker M. Small normal-metal loop coupled to an electron reservoir. Phys. Rev. B 32, 1846–1849 (1985). 10.1103/physrevb.32.1846 9937238

[b27] Cabib D. & Callen E. Charge order and antiferromagnetic order in the Hubbard model with nearest-neighbor Coulomb interaction: Weak coupling. Phys. Rev. B 12, 5249–5254 (1975).

[b28] Krishna R. P. M., Mukhopadhyay S. & Chatterjee A. Nature of the self-trapping transition in a one-dimensional Holstein– Hubbard model. Phys. Letts. A 327, 67–72 (2004).

[b29] Sankar I. V., Mukhopadhyay S. & Chatterjee A. Localization–delocalization transition in a two-dimensional Holstein–Hubbard model. Physica C 480, 55–60 (2012).

[b30] Sankar I. V. & Ashok Chatterjee Self-trapping phase diagram for the strongly correlated extended Holstein-Hubbard model in two-dimensions. Eur. Phys. J. B 87, 154–163 (2014).

